# Transcriptional and Microenvironmental Regulation of Lineage Ambiguity in Leukemia

**DOI:** 10.3389/fonc.2017.00268

**Published:** 2017-11-06

**Authors:** Tianyuan Hu, Rebecca Murdaugh, Daisuke Nakada

**Affiliations:** ^1^Department of Molecular & Human Genetics, Baylor College of Medicine, Houston, TX, United States; ^2^Program in Developmental Biology, Baylor College of Medicine, Houston, TX, United States

**Keywords:** lineage switch leukemia, mixed-phenotype acute leukemia, hematopoietic stem cells, acute myeloid leukemia, acute lymphoid leukemia, CAR-T cells

## Abstract

Leukemia is characterized by the uncontrolled production of leukemic cells and impaired normal hematopoiesis. Although the combination of chemotherapies and hematopoietic stem cell transplantation has significantly improved the outcome of leukemia patients, a proportion of patients still suffer from relapse after treatment. Upon relapse, a phenomenon termed “lineage switch” is observed in a subset of leukemia patients, in which conversion of lymphoblastic leukemia to myeloid leukemia or *vice versa* is observed. A rare entity of leukemia called mixed-phenotype acute leukemia exhibits co-expression of markers representing two or three lineages. These two phenotypes regarding the lineage ambiguity suggest that the fate of some leukemia retain or acquire a certain degree of plasticity. Studies using animal models provide insight into how lineage specifying transcription factors can enforce or convert a fate in hematopoietic cells. Modeling lineage conversion in normal hematopoietic progenitor cells may improve our current understanding of how lineage switch occurs in leukemia. In this review, we will summarize the role of transcription factors and microenvironmental signals that confer fate plasticity to normal hematopoietic progenitor cells, and their potential to regulate lineage switching in leukemias. Future efforts to uncover the mechanisms contributing to lineage conversion in both normal hematopoiesis and leukemia may pave the way to improve current therapeutic strategies.

## Introduction

Hematopoietic stem cells (HSCs) establish and maintain the hematopoietic system through differentiation into the multi-lineage progenitors and committed progenitors from which all the mature lineage cell types arise. In the classical model of hematopoiesis, long-term HSCs, short-term HSCs, and multipotent progenitors (MPPs) reside at the apex of the hierarchy (1–5). MPPs are able to differentiate into lineage-committed progenitors, including common lymphoid progenitors (CLPs) (6) and common myeloid progenitors, which further differentiate into granulocyte-monocyte progenitors (GMPs) and megakaryocyte-erythroid progenitors (MEPs) (7). A characteristic feature of this model is that, as progenitors differentiate through this pathway, their developmental potential narrows. For example, MEPs lack the granulocyte-monocyte (GM) potential of GMPs and instead have differentiation potential that is restricted to the megakaryocyte and erythroid (Meg/E) lineages. Importantly, although many studies have provided evidence supporting this classical hierarchy, studies have shown that the committed state can be canceled or reprogramed by the action of lineage specifying cytokines and transcription factors. This raises the question to what extent the committed states are fixed, and whether or not oncogenic mutations exploit the lineage promiscuous state of normal progenitor cells to change their phenotypes upon therapies.

## Lineage Commitment and Switch in Normal Hematopoiesis

**Figure 1:**
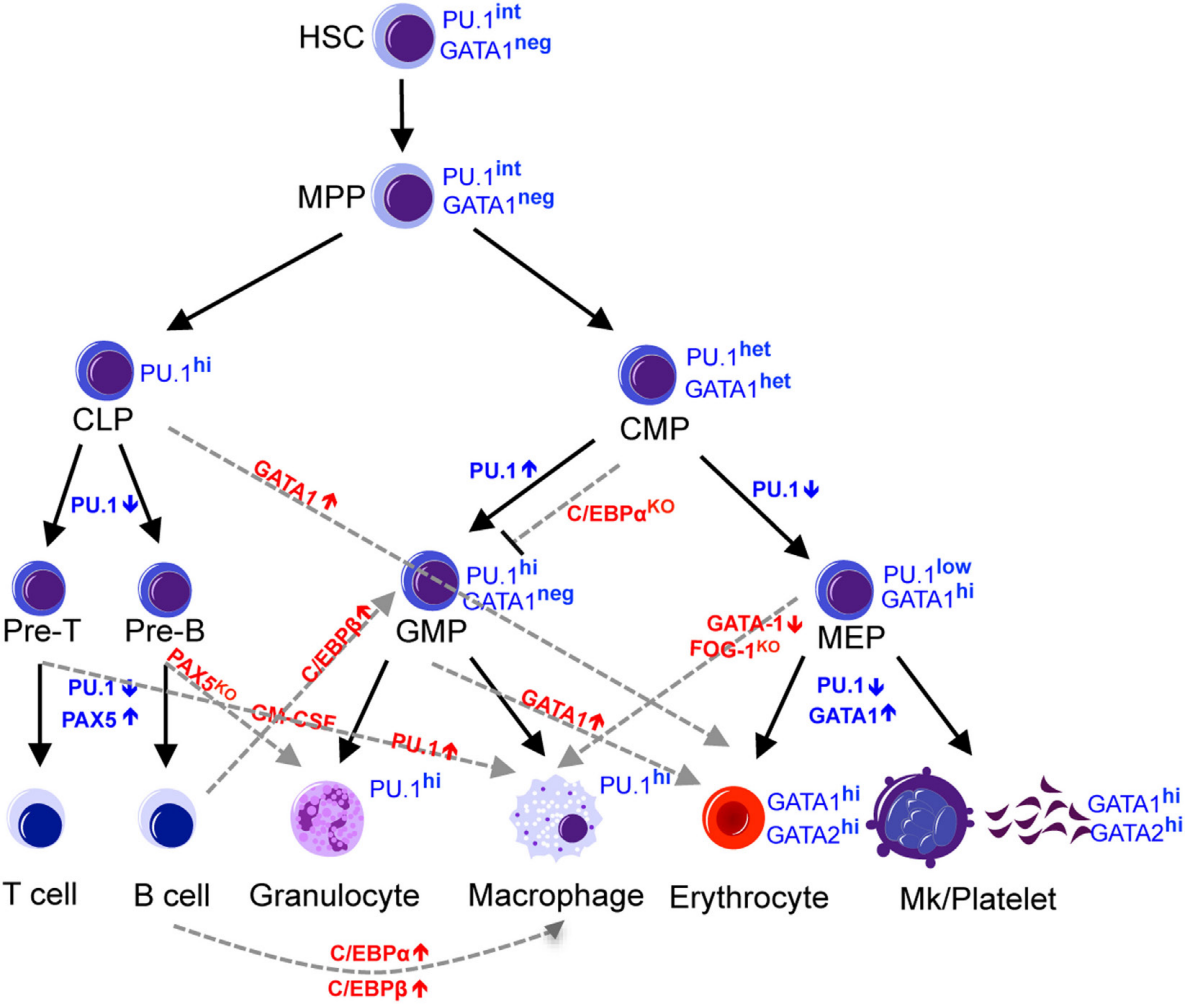
Regulators of lineage commitment in normal hematopoiesis. The hematopoietic system is maintained by HSCs, which gradually lose developmental potential through differentiation into downstream progenitors and mature cells. This narrowed lineage potential is controlled by a precise combination of transcription factors and can be reprogrammed by manipulating the expression level of certain transcription factors. Black solid lines indicate the normal commitment steps, while the gray dashed lines indicate altered lineage potential by manipulating the levels of transcription factors. HSC, hematopoietic stem cells; MPP, multipotent progenitors; CLP, common lymphoid progenitors; CMP, common myeloid progenitors, MEP, megakaryocyte-erythroid progenitors; GMP, granulocyte-monocyte progenitors.

The increasingly narrowed lineage potential results from a precise combination of gene expression signatures and epigenetic modification. Several transcription factors have been found to be involved in the fate decision of hematopoietic progenitors (8, 9). Among these are the lineage-specific master regulator transcription factors, such as Pu.1 (also known as Spi-1; spleen focus forming virus proviral integration oncogene 1), C/ebp-α, Gata1, Pax5, and Ikaros (Figure 1). Pu.1 and C/ebp-α are master regulators of the myeloid cell fate, and not only do these transcription factors promote myeloid differentiation of progenitor cells (10) but also ectopic expression of these transcription factors confer a myeloid cell fate to cells of other lineages, such as T-cells, B-cells, or fibroblasts (11–14). Gata1 is a master regulator of erythroid cell fate that is required and sufficient to confer the erythroid fate. Deletion of Gata1 in mice causes defective erythropoiesis (15–21), whereas ectopic expression of Gata1 confers Meg/E fate to cells of other lineages, such as monocytic cells (22, 23). Loss of B-cell master regulators Pax5 and Ikaros disrupts the B-cell transcriptome and reprograms B-cells into myeloid (24, 25) or epithelial-like cells (26), respectively. Lineage conversion by ectopic expression or loss of these master regulator transcription factors is often associated with a change in a network of transcription factors that governs cell fate. Pu.1 promotes multipotent hematopoietic progenitors to differentiate into myeloid cells by activating multiple myeloid-lineage-related genes, including C/ebp-β and suppressing erythroid factors such as Gata1 (10), while C/ebp-α and C/ebp-β converts differentiated B-cells into macrophages by inhibiting Pax5 (12). The extent to which these transcription factors over-ride the lineage-committed state of differentiated cells illustrates the extensive potential these master regulators possess.

## Lineage Ambiguity in Malignant Hematopoiesis

Leukemias develop as a consequence of mutations that cooperatively confer aberrant self-renewal capacity to leukemic cells and allow them to proliferate indefinitely without differentiation. Recent advances in high-throughput sequencing of leukemia genomes have revealed numerous mutations in cytokine signaling, epigenetic regulators, and transcription factors (27, 28). Genetic studies using murine models have established that mutations in epigenetic and transcriptional regulators upregulate self-renewal and block differentiation of hematopoietic stem/progenitor cells (HSPCs) (29). The ability of transcription factor levels and external cytokine milieu to influence hematopoietic progenitor plasticity raises the question of whether lineage conversion plays any role in malignant hematopoiesis that often carry mutations in these regulators. In fact, a phenomenon called lineage switch has been reported, in which patients with acute leukemia that meet the French–American–British classification for being lymphoid or myeloid leukemias relapse with acute leukemia of the other lineage. Most cases of lineage switches are from ALL to AML (30–34), but AML to ALL switches have also been reported (33, 35–37). Additionally, some leukemias show no clear evidence of differentiation into a single lineage. These leukemias, termed mixed-phenotype acute leukemia (MPAL) (38) exhibit cells of at least two lineages; MPALs involving B-cell and myeloid lineages are the most frequent but some rare cases involve B- and T-cells, or B/T/myeloid cells. Patients with lineage switch leukemia or MPAL have poor prognosis, due to the difficulty in diagnosis and the lack of set protocols to guide treatments (38–41). Understanding the molecular mechanism behind lineage switch and ambiguity should pave a way for better treatment. We will discuss several hypotheses that have been proposed to explain the lineage switch and ambiguity in leukemias. These mechanisms are not mutually exclusive and likely occur in parallel. For example, dysregulation of lineage-specific transcription factors may generate an aberrant bi-potential leukemic clone, and therapies may facilitate the selection of bi-potential clones that are better equipped to survive the therapy by changing their phenotype.

### Multipotency of Leukemic Clones

One potential mechanism to explain how some leukemias switch their lineages is that these leukemias were derived from bi-potential clones. When leukemia cells from a patient who exhibited T-cell acute lymphoblastic leukemia (T-ALL) to AML switch upon chemotherapy were transplanted into SCID mice, engrafted AML cells exhibited myeloid cell markers (such as CD33) as expected, but the cells exhibited T-cell markers (such as CD2, CD4, and CD7) similar to the T-ALL at diagnosis if the recipient mice were treated with cytokines GM-CSF or interleukin 3 (IL-3) (42). Interestingly, although a common NRAS mutation was identified at every time point during the study (T-ALL at diagnosis, AML upon lineage switch, and T-ALL or AML in SCID mice) suggesting that both T-ALL and AML were derived from a common founding clone, the TCR rearrangement observed in T-ALL at diagnosis was not observed in patient’s AML cells upon switch nor the cells in SCID mice. This results indicate that the AML emerged in the patient upon lineage switch were not derived from T-ALL cells with TCR rearrangement, and suggests that a common *NRAS* mutated bi-potent leukemia clone with T-ALL and AML potential existed (42).

On the other hand, other studies have shown the presence of TCR rearrangements in myeloid leukemia cells upon lineage switch from lymphoid leukemias (43, 44). In these cases, it remains unclear whether the lymphoid leukemia clones with TCR rearrangements had bi-potential at diagnosis, or whether the lymphoid clones gained myeloid potential through potential mechanisms discussed below. Similarly, reports on B-cell precursor acute lymphoblastic leukemia (BCP-ALL) to AML switch have suggested that a bi-potential B-myeloid progenitor, which has been detected in fetal and adult mice (45, 46), may have become transformed, but evidence that such bi-potential progenitor cells are the origin of the disease and existed at diagnosis is lacking. It is equally possible that a B-ALL clone changed the phenotype upon treatment due to selective pressure.

### Clonal Selection and Therapies

**Figure 2:**
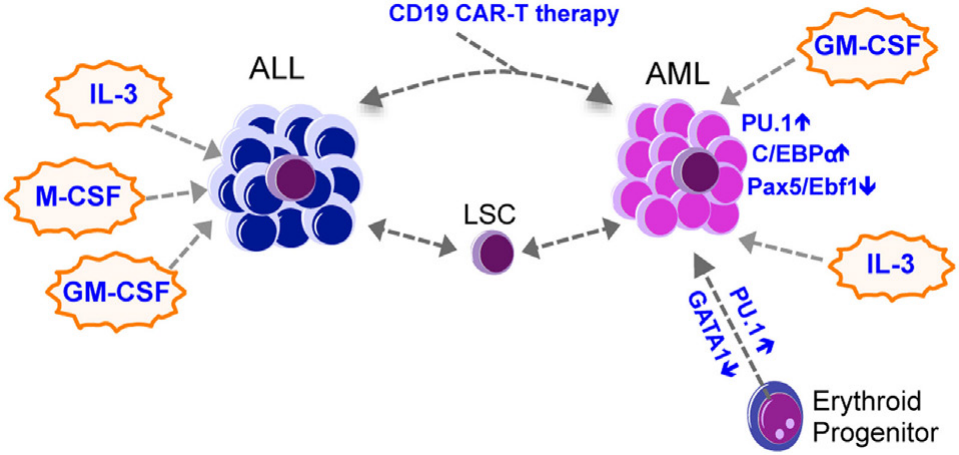
Lineage switch in leukemia. Most lineage switch in leukemia is from ALL to AML, with occasional AML to ALL switches being observed. Lineage switch could be mediated by therapy-mediated (such as CAR-T immunotherapies) selection of heterogeneous leukemic clones that survive the therapy and expand to produce an altered lineage output. Transcription factors such as Pu.1, C/ebp-α, Pax5, and Ebf1, together with cytokines such as IL-3, M-CSF, and GM-CSF, may mediate the lineage switch. AML, acute myeloid leukemia; ALL, acute lymphoid leukemia; CML, chronic myeloid leukemia; LSC, leukemia stem cell; GM-CSF, granulocyte-macrophage colony-stimulating factor; M-CSF, macrophage colony-stimulating factor; IL-3, interleukin 3.

Lineage switch is often associated with therapy relapse, suggesting that clones with altered phenotypes emerge as a consequence of the selective pressure imposed by the therapy. Therapies can eradicate the dominant clone(s) but select for a latent clone that survived the therapy, or the dominant clone may acquire additional mutations to evolve. While this has been elegantly demonstrated in relapsed AML (47), therapy-related selection has also been reported in lineage switched leukemia. For example, Podgornik et al. reported a B-ALL patient who relapsed with AML from a separate clone that survived the B-ALL therapy (48), while Mantadakis et al. reported on a pediatric patient with T-ALL who relapsed with AML after chemotherapy (49). Recent findings with B-ALL immunotherapy further lend insight into this mechanism (Figure 2). Chromosomal rearrangements at 11q23 are found in both AML and ALL and result in the fusion of the MLL1 gene with approximately 80 partner genes, among which *AF4* is the most common partner (50). MLL–AF4 fusion is mostly associated with pro-B-ALL expressing some myeloid cell markers such as CD15 (51). Recent studies reported cases of MLL–AF4 rearranged pro-B-ALL patients treated with blinatumomab, a bispecific antibody that targets CD19 on B-cells, who relapsed with AML (52–54). Transplantation of the relapsed AML into NSG mice caused CD19^+^ B-ALL that was genetically related to the relapsed AML, suggesting that the anti-CD19 treatment selected for clones that downregulated CD19 and acquired phenotypic changes toward the myeloid lineage (52). Moreover, CD19 CAR-T therapy caused relapse accompanied by a phenotypic change from pre-B-ALL to a myeloid phenotype (55, 56). The authors also used a mouse E2a–PBX1 transgenic B-ALL model and demonstrated that mouse CD19 CAR-T treatment relapses by either causing an alternative splicing of CD19 in B-ALL cells to escape CAR-T cells, or by causing a switch to myeloid leukemias with low Pax5 and Ebf1 expression and increased expression of CD11b and Gr-1 (56). Relapsed myeloid leukemia cells were not detected in E2a–Pax5 pre-B-ALL cells using single cell approaches, suggesting that CAR-T unlikely selected for rare myeloid leukemia cells but instead reprogrammed the B-ALL cells into a myeloid fate. These findings reinforce the idea that some leukemias retain the plasticity to drastically change their phenotype in the face of a strong selection imposed by therapies, and that lineage switch might represent a novel mechanism of resistance against immune therapies.

### Cell Reprogramming by Transcription Factors

Similar to how lineage-specific transcription factors reprogram the fate of normal hematopoietic progenitors, these transcription factors have a large impact upon the fate of leukemia cells. Leukemogenic mutations appear to tip the balance created by the network of transcription factors to block and/or bias the differentiation program toward a particular lineage. Dysregulated expression of Pu.1, Gata1, and C/ebp-α all contribute to leukemogenesis (57), and some of these factors are used by leukemic fusion genes to promote aberrant self-renewal of leukemia. For example, AML caused by MLL-fusion genes or MOZ-TIF2 depends upon Pu.1 for their maintenance (58, 59). Recent studies also indicate that these lineage-specific transcription factors regulate the fate choice of leukemia, and may contribute to the lineage switch of leukemias observed upon therapies.

In zebrafish models, AML1–ETO upregulates Pu.1 and downregulates Gata1 to convert the fate of erythroid cells into granulocytic cells, causing a phenotypic change similar to human AML (60). Overexpression of Pu.1 was reported in a rare case of adult Philadelphia chromosome-positive bilineage leukemia (myeloid and T-cell), in which TCR rearrangement was detected in both the myeloid and T-cell compartments of the disease, suggesting that the AML population emerged from T-ALL cells, potentially due to Pu.1 expression (61). Similar to Pu.1, C/ebp-α is also found to be involved in leukemia lineage switch. Slamova et al. reported cases of BCP-ALL that underwent lineage switch upon therapy to monocytic leukemias, which carried the same Ig/TCR rearrangements as the original BCP-ALL (43). The monocytic leukemias with Ig/TCR rearrangements had C/ebp-α promoter hypomethylation accompanied by increased C/ebp-α expression compared to the original BCP-ALL (43). These results suggest that myeloid transcription factors, such as Pu.1 and C/ebp-α, are involved in promoting lymphoid leukemias to switch their fate to a myeloid fate. Additional evidence suggests that the lineage switch can be promoted by the loss of lymphoid transcription factor expression. In the reported case of CD19 CAR-T-induced lineage switch of B-ALL to AML (56), expression levels of lymphoid transcription factors Pax5 and Ebf1 were reduced. Mouse model of CAR-T-induced lineage switch also revealed epigenetic changes leading to loss of Pax5/Ebf1 and increase of C/ebp-α expression and demonstrated that deletion of Pax5 or Ebf1 promoted the lineage switch from B to myeloid fate without CAR-T therapy (56). However, it is still unclear to what extent myeloid transcription factors contribute to the switch toward the myeloid fate, and whether the lineage-specific transcription factors can be exploited to block switching or target the switched leukemias.

### Microenvironment

The local microenvironment of HSPCs regulates the maintenance of HSPCs and is often altered in hematological malignancies (62–64). The microenvironment influences disease initiation (65), progression (63), and the efficacy of the therapies (66) by modulating the cytokine milieu and the metabolic parameters. Moreover, similar to how lineage-instructing cytokines can affect the fate of normal HSPCs, leukemia cells with certain plasticity exhibit different lineage output depending on the cytokine milieu.

MLL-translocated leukemias appear to retain lineage plasticity that can be tapped to direct the differentiation toward either B-cell or myeloid lineages using different cytokines. Expression of MLL-fusion oncogenes in cord blood HSPCs induces B-ALL upon xenotransplantation (67). The types of leukemias these oncogenes caused was affected by the culture conditions, as MLL–ENL expressing cells that are prone to cause B-ALL initiated AML with rearranged IgH when cultured in myeloid-promoting conditions. The ability of MLL–AF9 oncogene to produce B-ALL or AML is also affected by the culture condition, as well as the humanized cytokines expressed in the recipient immunocompromised mice (68). The fusion product of human MLL and murine Af4 (MLL–Af4) initiates pro-B-ALL that recapitulates the human pathology but causes AML when the cells were culture in myeloid-promoting conditions (52). Interestingly, MLL–Af4 transformed myeloid cells cultured in a myelopoietic condition had increased expression of lymphoid regulators, such as Ebf1, compared to AML cells transformed by MLL–AF9, suggesting that the myelopoietic condition cannot fully rewire the lymphoid program imposed by MLL–Af4.

A recent study demonstrated that BCR–ABL1 rearranged B-ALL can be reprogrammed to a myeloid fate by myeloid-instructing and proinflammatory cytokines (69–73). Purified B-ALL blasts exhibited myeloid cell marker expression and phagocytotic phenotypes upon stimulation by IL-3, M-CSF, and GM-CSF. The reprogrammed macrophage-like cells (termed MLCs) had increased expression of myeloid master regulators C/ebp-α and Pu.1, and corresponding overexpression of C/ebp-α and Pu.1 significantly induced myeloid reprogramming, suggesting that the myeloid cytokines and myeloid transcription factors cooperate to confer a myeloid cell fate to B-ALL, consistent with the ability of these two transcription factors to confer a myeloid fate to lymphocytes (11, 12, 14). Interestingly, although the original B-ALL cells that failed to reprogram into MLCs had the ability to cause B-ALL in recipient mice upon xenotransplantation, the reprogrammed MLCs had negligible ability to engraft. Since lineage switch is often associated with relapse and worse clinical outcome, it is unclear whether promoting myeloid reprogramming can be used as a therapeutic strategy. Nonetheless, depending on how deeply leukemias can be directed to differentiate, instructing cells to differentiate into other lineages may provide a novel therapeutic option.

## Conclusion

Similar to normal hematopoietic cells, leukemia cells also exhibit lineage plasticity and reversibility aided by master transcriptional regulators that control lineage determination of normal hematopoietic progenitor cells, by the instructive cytokine milieu and also by the strong selective pressure imposed by therapies. Although some improvements in treatment outcome have been reported by the use of intensified ALL therapy followed by AML therapy upon lineage switch, treatment of these leukemia remains challenging (74, 75). The ability of B-ALL cells to change their lineage-specific cell surface marker expression in response to immunotherapies underscores the clinical challenges posed by the plasticity of leukemia. Molecular characterization of the fundamental requirements of leukemias may reveal new strategies to target the disease regardless of their lineages.

## Author Contributions

TH, RM, and DN reviewed the literature and wrote the manuscript. TH designed the figures.

## Conflict of Interest Statement

The authors declare that the research was conducted in the absence of any commercial or financial relationships that could be construed as a potential conflict of interest.
